# A genotype-specific, randomized controlled behavioral intervention to improve the neuroemotional outcome of cardiac surgery: study protocol for a randomized controlled trial

**DOI:** 10.1186/1745-6215-14-89

**Published:** 2013-04-01

**Authors:** Daniela Hauer, Iris-Tatjana Kolassa, Rüdiger Paul Laubender, Ulrich Mansmann, Christian Hagl, Benno Roozendaal, Dominique J-F de Quervain, Gustav Schelling

**Affiliations:** 1 Department of Anaesthesiology, Ludwig-Maximilians University, Munich 81377, Germany; 2 Clinical and Biological Psychology, Institute of Psychology & Education, University of Ulm, Ulm 89069, Germany; 3 Institute of Medical Informatics, Biometry, and Epidemiology, Ludwig-Maximilians University, Munich 81377, Germany; 4 Department of Cardiac Surgery, Ludwig-Maximilians University, Munich 81377, Germany; 5 Department of Cognitive Neuroscience, Radboud University Nijmegen Medical Centre and Donders Institute for Brain, Cognition and Behaviour, Radboud University Nijmegen, Nijmegen, 6500 HB Nijmegen, The Netherlands; 6 Division of Cognitive Neuroscience, University of Basel, Basel 4056, Switzerland

**Keywords:** Cognitive behavioral intervention, Genotyping, Glucocorticoid receptor, Personalized medicine, Post-traumatic stress disorder, Prophylaxis, Single nucleotide polymorphism

## Abstract

**Background:**

Cardiac surgery is one of the most commonly performed surgical procedures worldwide with >700,000 surgeries in 2006 in the US alone. Cardiac surgery results in a considerable exposure to physical and emotional stress; stress-related disorders such as depression or post-traumatic stress disorder are the most common adverse outcomes of cardiac surgery, seen in up to 20% of patients. Using information from a genome-wide association study to characterize genetic effects on emotional memory, we recently identified a single nucleotide polymorphism of the glucocorticoid receptor gene (the Bc*l*l single nucleotide polymorphism) as a significant genetic risk factor for traumatic memories from cardiac surgery and symptoms of post-traumaticstress disorder. The Bc*l*l high-risk genotype (Bc*l*l GG) has a prevalence of 16.6% in patients undergoing cardiac surgery and is associated with increased glucocorticoid receptor signaling under stress. Concomitant animal experiments have confirmed an essential role of glucocorticoid receptor activation for traumatic memory formation during stressful experiences. Early cognitive behavioral intervention has been shown to prevent stress-related disorders after heart surgery.

**Methods/Design:**

The proposed study protocol is based on the above mentioned earlier findings from animal experiments and preclinical studies in volunteers. Patients (n = 872) will be genotyped for the Bc*l*l single nucleotide polymorphism before surgery, which should result in 120 homozygous high-risk carriers of the Bc*l*l GG allele and 240 randomly selected low-risk heterozygous or non-carriers of the single nucleotide polymorphism. All patients will then undergo randomization to either cognitive behavioral intervention or a control intervention consisting of non-specific general information about the role of stress in heart disease. The primary efficacy endpoint will be post-traumatic stress levels at one year after surgery as determined by a standardized questionnaire that has been specifically validated in patients after critical illness.

**Discussion:**

The proposed randomized controlled trial intends to demonstrate that a preoperatively administered minimal cognitive behavioral intervention targeted to homozygous carriers of the Bc*l*l *G high-risk allele reduces traumatic memories and post-traumatic stress disorder symptoms after heart surgery to a level seen in non-carriers of the mutation, and thus improves the neuroemotional outcome of cardiac surgery.

**Trial registration number:**

The trial will be registered at http://www.clinicaltrials.gov/ before commencing with the study.

## Background

Cardiac surgery (CS) is one of the most frequent surgical procedures worldwide with more than 700,000 cardiovascular procedures performed in 2006 in the US alone [1]. Surgery and the subsequent intensive care unit (ICU) therapy represent a considerable physical and emotional stressor [2]. Whereas physical functioning after CS improves over time, mental disorders as a result of high perioperative stress such as chronic anxiety, depression or post-traumatic stress disorder (PTSD) can persist for months and years [2,3]. Up to 20% of patients develop PTSD symptoms after CS [3]. Post-traumatic stress in the presence of heart disease is linked to impaired health-related quality of life [4], reduced rate of return to professional activities, increased health-care utilization with considerable cost for health-care systems [5] and a poor long-term cardiovascular prognosis [6]. However, although efficient treatments of PTSD exist, little is known about preventing PTSD and other stress-related disorders through evidence-based interventions in critically ill patients undergoing CS or intensive care therapy for other reasons. Therefore, targeted and cost-effective interventions to improve the neuroemotional outcome of extensive medical procedures are urgently required [7].

In this randomized controlled trial, we intend to use an innovative and personalized approach by identifying carriers of a genetic risk factor (Bc*l*l *G) for chronic stress after CS and investigate whether a targeted minimal behavioral intervention can counteract this genetic vulnerability for PTSD symptoms and improve long-term mental health.

### The Bc*l*l SNP of the glucocorticoid receptor influences traumatic memories and PTSD stress symptoms in patients undergoing cardiac surgery

**Figure 1:**
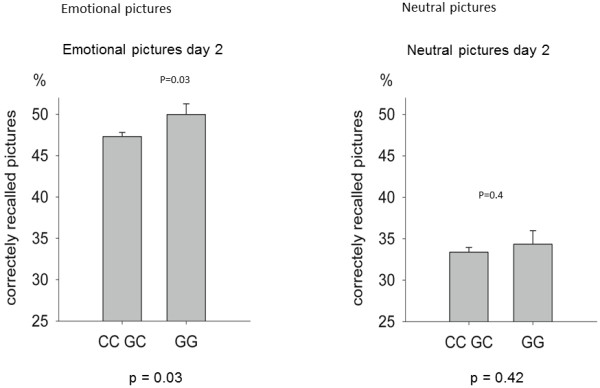
**Percentage of correctly recalled emotional pictures as compared to neutral pictures as a function of the Bc*l*l single nucleotid polymorphism of the glucocorticoid receptor gene in a genome-wide association study in 842 healthy individuals.** Homozygous GG carriers of the single nucleotide polymorphism recalled more emotional pictures than heterozygotes or non-carriers (left graph). The single nucleotide polymorphism did not influence the recall of neutral pictures (right graph). Unpublished data provided by DQ.

**Figure 2:**
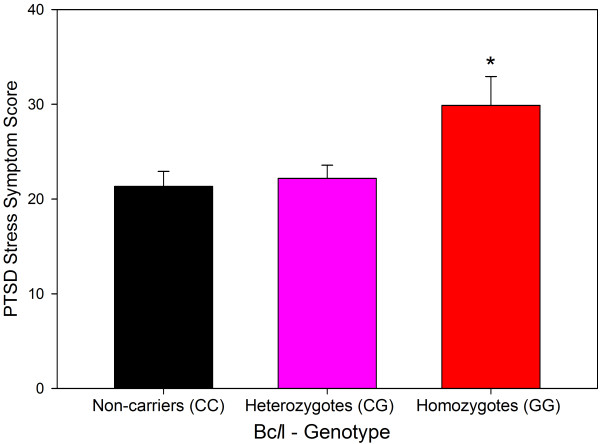
**PTSD stress symptom scores according to Bc*l*l genotype one week after discharge from the intensive care unit in a recent confirmatory study in patients undergoing cardiac surgery (n = 95).** *P = 0.02 compared to non-carriers (CC) of the SNP (one-way analysis of variance with Holm-Sidak post-hoc test). Data represent means ± standard error of the mean (unpublished). PTSD, post-traumatic stress disorder.

The major risk factor for PTSD development in critically ill patients is the presence of traumatic memories from highly stressful experiences during ICU treatment [2,8]. Animal experiments performed by our research group and others have repeatedly shown that the encoding of traumatic experiences into emotional memory during stress is facilitated by a stress-induced increase in glucocorticoid [9] and endocannabinoid signaling [10]. Using information from a genome-wide association study to characterize genetic effects on emotional memory in volunteers (Figure 1), we have recently identified an intronic SNP of the glucocorticoid receptor (GR) gene (the Bc*l*l SNP) in patients undergoing CS that is associated with increased cortisol sensitivity of the GR and, thus, enhanced glucocorticoid signaling under stress. The increased cortisol sensitivity is seen in homozygous carriers of the Bc*l*l *G allele, which has a prevalence of 14% in the western European population [11] and 16.6% in our patients undergoing CS [12]. Homozygous carriers had a significantly increased risk for traumatic memories and PTSD symptoms of chronic anxiety, pain, depression and impaired health-related quality of life after CS [12]. We very recently confirmed this finding in a second study in an independent group of patients having CS (unpublished, Figure 2). In combination, these well-corroborated findings allow the preoperative identification of patients at risk for PTSD symptoms after heart surgery who could benefit from preventive interventions.

### A cognitive behavioral intervention is effective in preventing PTSD symptoms after intensive care therapy

Although counterintuitive, increased analgesia and sedation of critically ill patients intended to prevent traumatic experiences during ICU treatment has not been successful in avoiding PTSD but has shown the opposite effect [13]. By contrast, a number of early interventions based on cognitive behavioral intervention (CBI) have been effective in improving the neuroemotional outcome of critical illness. A study in patients having CS showed that preoperative CBI (for example, explaining cardiac myths and misconceptions, detailing what to expect during the hospital stay and subsequent recovery period) improved physical function and depression after surgery [14]. Early psychological intervention in patients during ICU treatment by trained psychologists resulted in a lower incidence of PTSD symptoms and a lower use of psychotropic medication during follow-up [15]. The use of an intensive care diary, which has comparable effects to CBI by changing how patients think about their illness as they reread the story and build an autobiographical memory, has also been effective in reducing PTSD symptoms in patients after ICU therapy [16]. A detailed explanation of the forthcoming events during CS and ICU care will enhance the predictability of the events and thereby reduce feelings of anxiety, helplessness and life-threat. Evidence from both animal [17] and human [18] research clearly indicates the detrimental effects of unpredictable stress, whereas anticipated stress exhibits a milder impact on both psychological and physical health. A preoperative CBI enhances predictability and should therefore reduce the arousal during and directly after surgery in the ICU and, consequently, the release of excessive amounts of stress hormones and endocannabinoids (ECs) [10] that contribute to the hyperconsolidation of this potentially traumatic situation. Secondly, prominent theoretical models of PTSD propose a dissociation between hyperconsolidated emotional memories and poorly consolidated autobiographical context memories as the root cause of intrusive memories in PTSD [19,20]. Therefore, a preventive CBI should provide detailed context information prior to CS and shortly after CS, to locate potential traumatic experiences in time and space. Although previous studies have demonstrated that CBI has benefits in improving the neuroemotional outcome of critical illness, none of the earlier investigations were aimed specifically at patients with a genetically identified risk. By contrast, we hypothesize that a preoperatively administered minimal behavioral intervention targeted to carriers of a genetic risk factor (Bc*l*l GG) will reduce the risk for development of PTSD to that of non-carriers of the allele. This approach would be analogous to a prophylactic immunization of individuals with a well-characterized risk for an infectious disorder.

## Methods/Design

### Intervention scheme/trial flow

**Figure 3:**
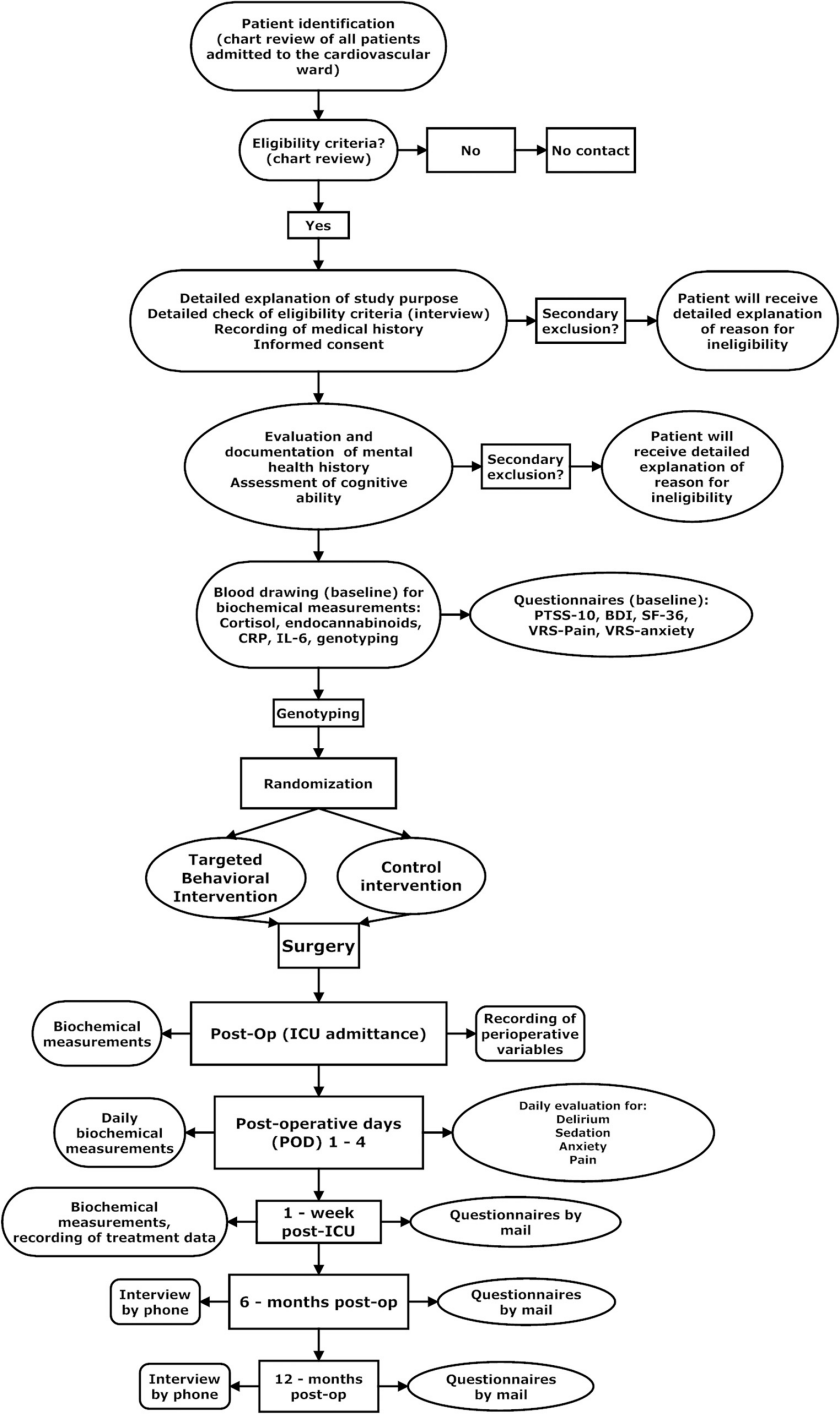
**Flow chart showing the sequence of events and key points of decision making during the proposed study.** Rounded rectangles indicate tasks to be performed by medical personal (for example, the study physicians). Ovals represent the responsibilities of a study psychologist and his or her team.

The flow of events during the study is depicted in Figure 3. After admission to the cardiac surgical ward, patients eligible for the study will be asked whether they are willing to participate. Patients will be told that if they agree to take part in the study, they can expect the following to take place. They will undergo genetic testing to identify a genetic risk within the stress hormone system for chronic stress reactions after surgery. They will then be randomized to either Intervention A, which provides specific information on postoperative procedures and focuses on possible and unexpected stressful experiences while in the ICU (the minimal behavioral intervention), or to Intervention B, which gives more general information about the role of stress during the perioperative period of CS.

After providing written informed consent, blood drawing for genotyping and an evaluation of cognitive ability will follow using the Mini Mental State Examination. Before randomization to either the intervention or the control group, the patients will be asked to complete a set of validated questionnaires to evaluate PTSD stress symptoms, standardized disease-related traumatic memories, depression, health-related quality of life, and current anxiety and pain. Randomization will be followed by the intervention, performed by a psychologist. After surgery, the patients will be admitted to the cardiovascular ICU. While in the ICU (postoperative days 1 to 4), the patients will be evaluated every 8 hours for sedation, delirium, pain and anxiety. Treatment variables will be recorded. One week after discharge from the ICU, the patients will be approached again by the study psychologist and asked to complete the set of questionnaires on traumatic memories from the ICU and post-traumatic stress intensity as well as current anxiety and pain symptoms. Six and 12 months after surgery, the patients will be interviewed by phone and will be sent the set of questionnaires by mail.

The primary efficacy endpoint of the study is a reduction in post**-**traumatic stress measured by questionnaire 12 months after surgery. The primary hypothesis to be tested is that this reduction is more pronounced for high-risk homozygous Bcll SNP carriers compared to the remaining patients.

### Data collection

Collected data will include demographic factors, objective indicators of myocardial function and severity of coronary artery disease, current pharmacologic treatment and dosage, cardiovascular risk factors, medical comorbidities, mental health history, episodes of high stress exposures related to the current hospitalization, recurrent symptoms of coronary heart disease, recurrent hospitalizations, compliance with medication, and type and intensity of rehabilitation after CS.

### Inclusion criteria

•Planned CS for coronary artery bypass grafting or valvular heart disease using cardiopulmonary bypass

•age >18 years

•sufficient knowledge of the German language in written and oral comprehension

•Western European origin (according to information provided by the patient).

### Exclusion criteria

•CS involving cardiocirculatory arrest under controlled hypothermia

•unstable preoperative cardiovascular condition requiring emergency procedures

•severe alcohol or drug abuse

•major pre-existing neurologic or psychiatric diseases (for example, PTSD, major depressive/bipolar or somatoform disorder)

•previous CS or treatment in an ICU with the exception of brief stays in a coronary care unit

•cognitive impairment as determined by the nine-item short version of the Mini Mental State Examination

•chronic organ dysfunction (renal, liver or lung)

•severe comorbidities likely to cause death within one year (for example, cancer)

•preoperative use of glucocorticoids (for example because of rheumatism)

### Selection of study population

The effect of the Bc*l*l SNP on post-traumatic stress and cortisol signaling has been characterized in patients undergoing CS at our institution who fulfilled the abovementioned criteria [12]. Patients satisfying the entry criteria of our study are in every aspect comparable to patients attending other European CS centers [21]. In addition, our study is a multi-center study with two centers (Klinikum Grosshadern and Herzklinik Augustinum, both in Munich). One can therefore assume that the results of our suggested study can be generalized.

#### Gender effects

**Figure 4:**
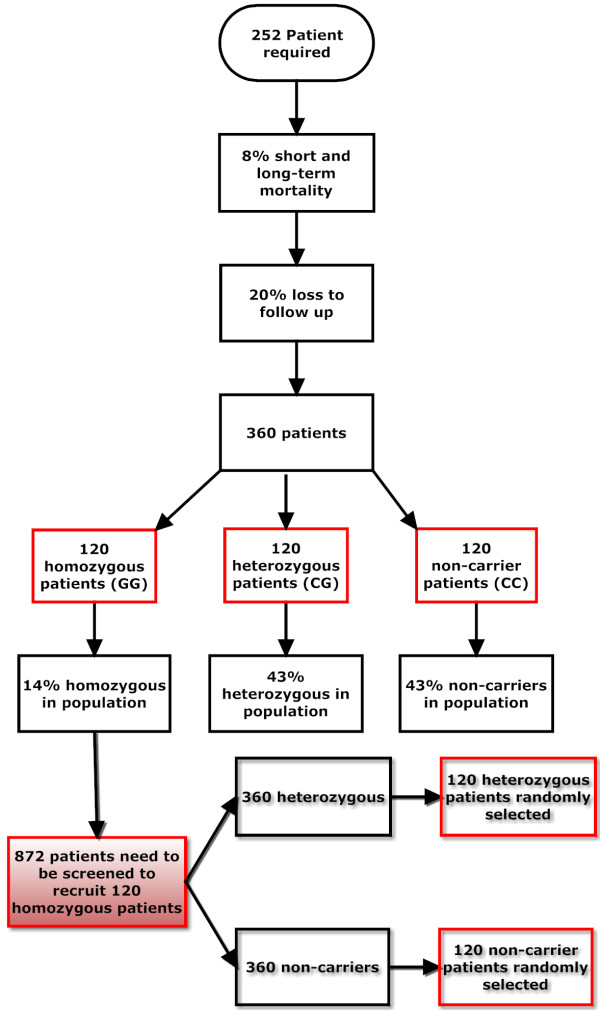
Flow chart depicting sample size estimation for the proposed study.

The selected study population addresses possible gender effects. In our previous study on the Bc*l*l SNP effect in patients undergoing CS, 30 of 126 patients studied were female. Of these 30 women, 6 were homozygous and 24 were heterozygous or non-carriers of the SNP [12]. Even in this small subgroup, female homozygous carriers had significantly higher PTSD scores one week after discharge from the ICU than female heterozygous non-carriers (29.0 ±13.1 versus 16.2 ±6.7, *P* = 0.009). In the proposed study, out of 180 patients intended to be included (Figure 4), approximately 43 will be female. Assuming that the effect of the homozygous Bc*l*l *G SNP and the gender distribution in both study samples is identical, our study will be adequately powered to allow the detection of gender-specific effects of the intervention on post-traumatic stress one week after ICU discharge and at one year (primary endpoint).

### Behavioral intervention to target traumatic memories and post-traumatic stress after surgery and intensive care unit treatment

The planned trauma-centered intervention will have an informative and resource-oriented approach to activate individual patient resources and cognitive (re)structuring to prepare for the oncoming surgery and subsequent ICU therapy. The major aim of the proposed intervention is to create realistic expectations about potential traumatic experiences that might occur, in order to render those experiences less surprising and hence reduce the subjective feeling of helplessness and distress in the patients. The intervention group will therefore be informed that some of them may have uncomfortable and potentially threatening experiences during the wake-up phase from anesthesia after heart surgery and while in the ICU. These potentially traumatic experiences could include nightmares and sometimes hallucinations or confusion, episodes of brief respiratory distress during weaning from mechanical ventilation, feelings of anxiety and some degree of pain from surgery. The study therapist will point out that these phenomena almost never occur during general anesthesia and heart surgery itself but are sometimes seen during the wake-up phase in the ICU after surgery. Participants will be told that these aversive experiences occur despite the best efforts of the therapeutic team in the ICU to avoid them by administering sedative and analgesic drugs in adequate dosages and the use of special techniques for extubation after mechanical ventilation. The therapist will also stress that these experiences usually do not indicate secondary complications after heart surgery and are generally self-limiting and of a temporary nature. The psychologist will further explore resources of the patient (for example, strategies that the patient had already successfully implemented to reduce stress, their social support networks, and future hopes and perspectives) and will encourage the patient to focus on these resources during and after ICU therapy. This information will be given to the patients in both written and oral form using simple and easily understandable language. A second part of the intervention is the provision of information about the context at the time of awakening, to orientate the patient in time and space directly after recovery from anesthesia. Likewise, the connection between sensory recollection about the potential traumatic experiences and autobiographical memories should be encouraged.

### Control intervention focusing on general aspects of psychological stress

To achieve significant differences between the trauma-focused ‘verum’ and the non-specific control intervention, the approaches need to be sufficiently different to avoid overlapping effects. The control intervention will therefore strictly avoid addressing the topics of ‘traumatic experiences or memories’ or ‘post-traumatic stress’ and, in contrast to the intervention group, no detailed information about the context of awakening will be given. Instead, the control group will receive information about the role of stress in heart disease and the possible benefits resulting from the use of stress management techniques in improving mental outcomes of CS. The control intervention will address questions regarding differences between normal daily stressors and pathological stress with dangerous health consequences, and why some individuals are more vulnerable to stress than others. It will also describe the relationship between pathological stress and an unhealthy lifestyle that results in an increased risk for heart disease. Furthermore, effective methods to reduce stress will be described to the patients. The patients will receive this information in both oral and written form. As during the specific intervention, all information will be given in a standardized and easily understandable manner.

### Additional treatments

All patients will be treated according to a strictly standardized protocol that has been used in all our previous studies addressing traumatic memories and PTSD symptoms after CS [2,12,22]. The use of the sedative agent propofol will be avoided, however, because a previous animal study by our group has shown that this compound may facilitate memory consolidation under stress by interacting with the endocannabinoid system [23]. Likewise, the administration of stress doses of hydrocortisone will also be avoided as hydrocortisone may have a preventive effect on the development of PTSD symptoms after CS [24].

### Outcome measures

#### Primary endpoint

We intend to use stress symptom scores determined by the German version of the modified Post-Traumatic Stress Symptom 10 questionnaire (PTSS-10) at one year after CS as the primary endpoint. The PTSS-10 instrument has been repeatedly validated in patients after ICU treatment. In the first validation study performed by our group, we used a double-blind interview technique performed by psychiatrists, according to the Diagnostic and Statistical Manual of Mental Disorders (DSM), third edition criteria, to diagnose PTSD. This validation process demonstrated a high internal consistency (Cronbach’s α = 0.93) and a high test-retest reliability (intraclass correlation coefficient *r* = 0.89) of the instrument. The specificity and sensitivity for diagnosis of PTSD after ICU treatment at a cut-off value of 35 points of the questionnaire were 97.5% and 77%, respectively [25]. The second and more recent validation study was performed by an independent group and according to the newer DSM-IV criteria and demonstrated that a PTSS-10 score of >20 one week after weaning from mechanical ventilation reliably identified patients who were diagnosed with PTSD three months later: sensitivity 100%; specificity 76% with an area under the receiver-operating characteristic curve of 0.91 [26]. The questionnaire has been used in multiple studies investigating traumatic memories and post-traumatic stress after ICU treatment by our [2,25,27-30] and other [15,16,31-34] groups and has also been shown to be sensitive to a pharmacologic intervention aimed at reducing PTSD stress symptoms after CS and ICU therapy in general, namely the use of hydrocortisone [24,33]. When used in patients with coronary artery disease alone, without the stressor of recent CS and at a single time point, the instrument appears to considerably overestimate the incidence of full syndromal PTSD when compared with an interview technique (Structured Interview for DSM Disorders). But even when used under conditions for which the instrument has not been specifically validated, the PTSS-10 was able to adequately quantify the intensity of disease-related stress symptoms [34].

This instrument consists of two parts: *Part A* evaluates four standardized categories of traumatic memory from the ICU and *Part B* quantifies the presence and intensities of 10 PTSD-related symptoms [25]*.*

##### Traumatic memories

A category of traumatic memory as measured by *Part A* of the questionnaire is defined as the patient’s subjective recollection of nightmares, respiratory distress/dyspnea, feelings of anxiety/panic or pain. When completing the questionnaire, the patient is asked to answer each of these four items ‘yes’ or ‘no,’ independent of the number of occasions the adverse experience occurred. The number of ‘yeses’ answered by a patient is termed the number of traumatic memories. The intensity of traumatic memories is not specifically evaluated by the questionnaire.

##### PTSD-related chronic stress symptoms

The severity and presence of PTSD symptoms is quantified by *Part B* of the questionnaire and evaluates the presence and intensity of ten PTSD-related stress symptoms: sleep disturbance, nightmares, depression, hyperalertness, withdrawal (emotional numbing and inability to care for others), generalized irritability, frequent changes in mood, guilt, fear and avoidance reactions with regard to the ICU, and increased muscle tension. When evaluating these symptoms, the patients are asked to think back over the last few days (‘presently - that means in the past few days - I suffer from…’), and then asked if they had symptoms expressed by statements such as: ‘Jumpiness, I am easily frightened by sudden sounds or sudden movements’ (to evaluate hyperalertness), ‘fear of places and situations which remind me of the intensive care unit’ (to evaluate avoidance reactions), or ‘a bad conscience, blame myself, have guilt feelings’ (to evaluate guilt). Patients rated their symptoms using a scale from 1 (never) to 7 (always). A summary score can then be calculated that ranges from 10 to 70 points, with increasing scores indicating a higher prevalence and intensity of PTSD symptoms.

Traumatic memories and PTSS-10 stress symptom scores will be evaluated before surgery, one week after discharge from the ICU and at six months and one year follow-up time points (Figure 3).

#### Secondary endpoints

##### Traumatic memories

Traumatic memories will be quantified by *Part A* of the modified PTSS-10 instrument (see above).

##### PTSD symptom intensity as measured by the Post-Traumatic Stress Diagnostic Scale

The Post-Traumatic Stress Diagnostic Scale is a brief but reliable self-report measure of PTSD symptoms for use in both clinical and research settings. This instrument will be used as a confirmatory measure of stress symptom intensity [35].

##### Depression

We intend to use the German version of the Beck Depression Inventory, which is regarded as a standard instrument to evaluate depression in epidemiologic studies and to rate depression in patients with cardiovascular disorders. Depression will be quantified preoperatively as well as six months and one year after CS.

##### Delirium

Delirium is a common complication in patients undergoing CS and is associated with negative outcomes including cognitive decline, increased mortality and a higher of rate distress after ICU therapy, although the association with PTSD development is not entirely clear. The occurrence of delirium during ICU treatment will be monitored using the Confusion Assessment Method instrument, which is recommended as a standard tool for confusion assessment in the ICU (for time points see Figure 3).

##### Sedation/agitation

During ICU treatment, we intend to quantify the degree of sedation versus the occurrence of agitation by applying the widely used Richmond Sedation Agitation Score, which classifies the patients into three agitation categories: agitated, calm or sedated. The instrument will be administered every 8 hours while the patients are treated in the ICU (postoperative days 1 to 4).

##### Pain intensity

We will ask the patients to indicate how strong their current pain is by using a verbal rating scale where 0 = no pain and 10 = the worst pain imaginable. Pain intensity will be evaluated at every time point and every 8 hours during ICU treatment.

##### Degree of anxiety

We will ask the patients to indicate how strong their current anxiety is by using a verbal rating scale where 0 = no anxiety and 10 = the highest anxiety imaginable.

##### Health-related quality of life

Mental and physical quality of life including physical functioning will be evaluated using the German version of the self-administered 36-item Medical Outcomes Study Short Form Survey [36].

##### Endocannabinoids

By measuring plasma ECs (see Figure 3 for time points of these measurements), we want to show that homozygous carriers of the Bc*l*l *G SNP with enhanced GR sensitivity have lower EC signaling as a result of an increased inhibitory effect of glucocorticoids on the EC system and that this interaction can be influenced by the targeted behavioral intervention. This assumption results from a pilot trial that demonstrated increased circulating concentrations of ECs in CS patients with traumatic memories and PTSD symptoms [37], a larger study in individuals with PTSD from war and torture experiences [38] and decreased perioperative plasma levels of ECs in CS patients who developed depression after heart surgery [39]. Plasma concentrations of the ECs anandamide, 2-arachidonoylglycerol, palmitoylethanolamide, N-arachidonoyldopamine, arachidonoylglycine, oleoylethanolamide, stearoylethanolamide, 2-arachidonoylglycerolether, N-oleoyldopamine, docosatetraenoylethanolamide, dihomo-y-linoleoylethanolamide and virodhamine will be determined using a method based on high performance liquid chromatography-tandem mass spectrometry as previously described [39].

##### Cortisol

Morning plasma cortisol (8 am) will be determined using standard methods. Plasma cortisol will be measured because, in our previous study, homozygous carriers of the Bc*l*l SNP had significantly lower morning plasma cortisol levels [12]. Cortisol determination may allow us to study the effect of the intervention on plasma cortisol concentrations as a function of genotype. Low plasma concentrations of cortisol have been described in some but not all patients with PTSD and stress doses of cortisol may reduce the intensity of PTSD stress symptoms after CS [24].

### Genotyping and biochemical analysis

All analyses will be performed from venous blood collected in the appropriate tubes at the time points indicated in Figure 3.

#### DNA preparation and genotyping

At least one day before surgery and after informed consent by the patients, 10 ml of venous blood will be collected and genomic DNA will be extracted using the QIAamp DNA Blood Maxi Kit (Qiagen, Hilden, Germany). Information on the Bc*l*l polymorphic site of the GR was derived from the database of single nucleotide polymorphisms established by the National Center for Biotechnology Information [40]. Genotyping will be done by high-resolution melting DNA analysis with specific probe sets. High-resolution melting represents a simple and low-cost solution with a short turnaround time, which is important for the preoperative genotyping intended in our study. Genotype distributions will be checked for violations of Hardy-Weinberg equilibrium.

#### Biomarkers of the systemic inflammation

C-reactive protein and interleukin-6 (IL-6) will be measured because patients with PTSD are known to have a tendency for inflammation and a chronic inflammatory response may mediate the increased cardiovascular risk seen in these patients. C-reactive protein will be determined preoperatively and daily thereafter until ICU discharge. For reasons of cost containment, IL-6 measurements will be limited to three time points: preoperatively, postoperatively and one week after ICU discharge.

### Statistical analyses

#### Sample size calculations

In several previous studies in CS patients, we assessed post-traumatic stress using the PTSS-10 instrument that will also be the primary outcome measure in this study. Our preliminary work indicated an 8-point difference on the PTSS-10 scale between homozygous Bc*l*l *G carriers as opposed to non-carriers [12]. This study also showed that the PTSS-10 score did not significantly decrease until six months after surgery. We may assume that the non-trauma-focused control intervention does not change post-traumatic stress levels in homozygous patients and that the difference of 8 points in post-traumatic stress scores between non-carriers and homozygous individuals is clinically meaningful. This assumption is supported by the recent study discussed in the section *Primary endpoint*[26] that showed that even a relatively low PTSS-10 score (>20) in patients one week after ICU therapy and weaning from mechanical ventilation was highly predictive for the later development of PTSD. Based on the results of our pilot study [12], we make the following assumptions: first, the mean PTSS-10 score one year after ICU discharge is 18±8 (mean ±SD) for non-Bc*l*l *G carriers and heterozygous Bc*l*l *G carriers and 25±8 for homozygous Bc*l*l *G carriers each under control intervention. Second, the mean PTSS-10 score one year after ICU discharge is 18 ±8 for all patients under experimental intervention. Both assumptions show a quantitative intervention-SNP interaction. An overall sample size of 252 patients (84 per SNP group) is needed assuming a type I error rate of 5% and a type II error rate of 80% to detect a statistically significant differential treatment effect between the genotype groups. The sample size calculation was performed by Nquery (Statistical Solutions Ltd.,4500 Airport Business Park, Cork, Ireland). Further, experience from former studies shows a perioperative mortality of 8% and a loss to follow-up of approximately 20% of patients at the six-month time point [2,12]. Adjustment for a drop-out rate of 30% leads to a total of 120 required patients (120–0.3 × 120 = 84) per SNP group. In our previous study in 126 Western European CS patients from our institution, 43% were non-carriers of the Bc*l*l *G, 43% were heterozygous Bc*l*l *G carriers and 14% were homozygous [12]. This means that we will have to genetically screen approximately 872 patients to detect 120 homozygous individuals eligible for randomization. Genetic screening will also result in the identification of approximately 376 non-carriers and 376 heterozygous individuals. These patients will also be randomly allocated to the control and the targeted intervention group but, to reduce costs, only 120 individuals with each genotype will be randomly selected by choosing every third consecutive heterozygous or non-carrier patient included in the study. Thus, we will have to screen and genotype 872 patients to include 360 patients into the final analyses (Figure 4).

#### Compliance rate and loss to follow-up

Previous studies from our group in the same study population using comparable criteria for study inclusion and the identical procedure for follow-up (phone calls and questionnaires) yielded a high rate of compliance and a low rate of loss to follow-up, which ranged between 20% [2] in 2003 and 16% in our most recent study [12]. The 16% figure was used in the sample size calculation for the project.

Statistically, appropriate imputation techniques based on the patient´s time course before dropping out will be used to handle missing values as a result of patient drop-out and recently suggested techniques for the prevention of patient drop-out will be observed [41].

#### Randomization

The randomization ratio to the control and intervention group is 1:1. Randomization will be performed by an internet-based tool (Randoulette) [42].

#### Primary analysis

A two-factorial analysis of variance model will be used that includes the binary factor ‘intervention’ and a trinomial factor ‘genotype’. In a first step, an ‘interaction’ between both factors will be assessed. If this interaction exists in a second step, differential treatment effects will be tested between the three pairs of genotype groups. The global alpha level is guaranteed since the closed testing procedure will be invoked [43]. The primary data analysis set is the intention-to-treat population, which analyzes the data of the patients as they will have been randomized [44].

#### Sensitivity analyses

The above analysis of variance model will be extended to a linear mixed-effects (LME) model by considering the longitudinal observations per patient (measurements at four time points).

#### Secondary endpoints

As the secondary endpoints are collected at four different times, we will use LME models (which respect the correlation of the observations within a patient evolving over time) and study the main and interaction effects of the intervention and the SNP groups on the (possibly adequately transformed) endpoints. Appropriate imputation techniques will be used to handle missing values (results of the LME model can help to impute the primary endpoint based on the patient’s time course before dropping out, points missing in the longitudinal data can also be imputed) [45]. The analysis will be performed using the statistical software environment R [46].

### Ethical considerations

The study has been approved by the Ethical Committee of the University of Munich (protocol# 137–11) and will be performed in accordance with the 1964 World Medical Association Declaration of Helsinki - Ethical Principles for Medical Research Involving Human Subjects in its current form from the 59th World Medical Association General Assembly, Seoul, October 2008. Further, the study will be conducted according to the *Guideline for Good Clinical Practice* (International Conference on Harmonization, Topic E 6 [R1]).

Due to the intended genetic testing, ethical aspects of the trial require special considerations. The participants will be informed that the research is performed solely for the purpose of generating scientific knowledge about genes or the genetic basis of disease and not to provide participants or their families with specific information about their genetic status or health, and at no time will this information be divulged by the investigator, without following specific guidelines provided. We will repeatedly stress that this study is for research purposes only and no individual results will be given back to the study participant, go into medical records or find their way to insurance companies or employers. This will include information from final results of the study, interim results of the study and incidental findings. The record of participation (including the consent form and research results) will never go into the medical record.

When giving informed consent the patients will receive the following information in both oral and written form:

a) The informed consent is for research purposes only and will *not* appear in the medical record.

b) Specific information resulting from genetic research will *not* be available to participants and their families.

c) Blood samples will eventually be shared with other investigators (third parties). Shared samples will be coded and remain anonymous to other investigators *and identification of patients from coded blood samples will be impossible for third-party researchers.*

d) Blood samples will eventually be used for secondary uses. In other words, research other than for what it was collected. Secondary uses will only be done on anonymized or anonymous samples.

e) A description of the special procedure required to allow re-contacting of patients or relatives despite strict confidence of genetic data.

f) Two copies of the informed consent will exist. An original copy will be filed in the research files and a second copy will be for the participant.

#### Potential benefits to the participants

All participants in our study will receive detailed and scientifically sound information regarding the relationship between stress and coronary heart disease and how to deal with possible stress effects after surgery. Thus, participation in our study may be associated with benefit for our patients, regardless of group assignment. Furthermore, we will remain in contact with our patients by phone and mail during the follow-up period of one year, which makes the detection of adverse outcomes feasible.

#### Methods of protecting participant privacy, data and rights

Every possible care will be given to issues of confidentiality. There will be no disclosure of study results to participants, family members, insurance companies, employers or other third parties without written permission of the participant and approval by the Ethical Committee of the University of Munich. Any shared samples will be anonymized to the recipient.

All participating patients will receive a unique four-digit identifier that will consist of a random combination of letters and numbers. The identifier will not contain letters or numbers derived from the patients name or date of birth. The patients names and addresses and the unique identifier will be stored in a coded file that will be kept separate from other data files generated during the study. All other files including those containing genetic data will use the four-digit identifier.

After termination of the study, all study records except genetic data will be stored in the central medical archive of the University Hospital of Munich but kept separate from medical records. A file linking genetic information with names and addresses of the patients will then be produced, a printout of this file will be stored by a notary and the original electronic version of the file destroyed. The notary will disclose the information allowing patient identification under two conditions: a written application by the original research team or the treating physicians of the patient or relatives describing the importance and possible benefit for patients or their relatives, and approval by the local ethical committee. Genetic information will only be disclosed to treating physicians or researchers but never to insurance companies, employers or other third parties.

### Safety

Study participation for the participants will be associated with minimal personal risk. Theoretically, the targeted intervention focusing on traumatic experiences and post-traumatic stress could result in a worsening of symptoms as has been described in individuals after involuntary emotional debriefing in the immediate aftermath of a highly traumatic event [47]. However, the fact that our planned intervention uses a cognitive and informational approach and is performed before possible emotional traumatization makes such negative effects very unlikely. The risks associated with blood sampling are also regarded as minimal. Nonetheless, the incidences of adverse events and serious adverse events in the treatment arms will be recorded by type and severity and follow-up will be provided by repeated phone calls.

## Discussion

Progress in the field of ICU therapy and medical procedures such as CS has resulted in improvements in short- and long-term survival rates in critically ill patients. This progress is, however, met by an in increasingly older population with increased life expectancy and multiple comorbidities requiring extensive surgical procedures and treatment in ICUs. By now, there are more than 10,000 critically ill patients treated in >1,500 ICUs on any given day in Europe [48] and CS is one of the most common surgical procedures performed worldwide. This imbalance between demand, disease severity and the required efforts for cost containment makes it unlikely that further progress in surgical performance and life support techniques in the ICU will result in meaningful improvements in long-term outcomes of critically ill patients with heart disease requiring surgery. There is increasing evidence that post-traumatic stress associated with critical illness and ICU therapy has long-lasting negative consequences on health-related quality of life, cognitive and social functioning, health-care utilization and even long-term mortality [2,49]. Published evidence also shows that behavioral interventions are able to prevent the development of post-traumatic stress symptoms related to exposure to a range of different malignant stressors including CS [14,50]. The overarching aim of the proposed project is to demonstrate that a targeted, CBI reduces the intensity of post-traumatic stress in patients undergoing CS and results in clinically meaningful improvements in long-term outcomes six and twelve months after surgery.

Time and staff resources of the therapeutic team caring for CS patients are limited, however, and the timely delivery of a targeted behavioral intervention to all CS patients in a busy tertiary care hospital may exceed the capacity of even large centers. Furthermore, the majority of patients do not develop stress-related disorders after CS and the intervention should therefore have no effect on outcome in these individuals. A preoperative identification of patients who might be particularly (or not at all) susceptible to the intervention is therefore highly desirable. The planned project addresses this issue and tests the novel approach of preoperative genetic testing to identify patients who might benefit from the intervention (in the sense of individualized, personalized or stratified medicine). If successful, the intervention could be tailored to patients at risk and a great number of CS patients might benefit with relatively little time investment. The added assessment of several biomarkers, both established (for example, cortisol, cytokines and C-reactive protein) and novel (for example, endocannabinoids), offers the additional benefit of giving information on intermediate biologic endpoint measures for traumatic memories, post-traumatic stress and the atherothrombotic process in relation to genotype.

We believe that the unique combination of genetic identification of patients at risk and testing the effect of a targeted behavioral intervention according to genotype on the neuroemotional outcome of CS is both sensible and innovative.

## Trial status

At the time of manuscript submission the study design has been evaluated by independent international reviewers and is under consideration for possible support by a major grant funder.

## Abbreviations

CBI: Cognitive behavioral intervention; CS: Cardiac surgery; DSM: Diagnostic and Statistical Manual of Mental Disorders; GR: Glucocorticoid receptor; ICU: Intensive care unit; IL-6: Interleukin-6; LME: Linear mixed-effects; PTSD: Post-traumatic stress disorder; PTSS-10: Post-Traumatic Stress Symptom 10 questionnaire; SNP: Single nucleotide polymorphism; WMA: World Medical Association.

## Competing interests

The authors declare that they have no competing interests.

## Authors’ contributions

DH performed the studies underlying the basic assumption leading to the study design and wrote part of the manuscript. IK and GS designed the study and drafted the manuscript. UM and RL produced the statistical analysis and the data management plan. CH provided essential data on cardiovascular procedures and outcomes at our institution. BR performed important behavioral studies characterizing the effects of glucocorticoid signaling on emotional memory under stress and DQ performed the molecular genetic studies and described the effects of the Bc*l*l SNP in healthy volunteers. All authors read and approved the final manuscript.
